# Multilevel correlates of domain-specific physical activity among rural adults – a cross-sectional study

**DOI:** 10.1186/s12889-022-14634-3

**Published:** 2022-11-23

**Authors:** Alan M. Beck, Natalicio H. Serrano, Audrey Toler, Ross C. Brownson

**Affiliations:** 1 Prevention Research Center, Washington University in St. Louis, One Brookings Drive, Campus Box 1196, St. Louis, MO 63130 USA; 2 Institute for Health Research and Policy, University of Illinois at Chicago, Chicago, IL USA; 3 Department of Surgery (Division of Public Health Sciences), Washington University School of Medicine, St. Louis, USA

**Keywords:** Physical activity domains, Rural, Adults

## Abstract

**Background:**

Increasing physical activity (PA) in rural communities is a vital prevention tactic in multiple chronic diseases; however, little is known on the multilevel correlates of PA rural areas. A better understanding of domain-specific PA adds context for promoting PA in rural communities. The current study sought to determine factors associated with domain-specific and overall moderate-vigorous physical activity (MVPA) in rural communities.

**Methods:**

Surveys were conducted across 14 rural mid-Western communities, with the final analytical sample including 1241 adults (ages 19–96, *M* = 57.0 [*SD* = 16.7], 67.8% female, 83.8% white). Generalized linear models with negative binomial distributions examined the relation between demographics, trail use, and perceptions of the neighborhood environment, with domain-specific and overall MVPA, measured via the Global Physical Activity Questionnaire.

**Results:**

Rural residents reported an average of 617 total minutes of weekly MVPA (SD = 1141), with 58.5% meeting MVPA guidelines. Higher age, female gender, and higher educated individuals had lower levels of overall and occupational MVPA. Females, higher education, and perceived indoor recreational access were associated with lower levels of transportation-related MVPA, while trail use was associated with increased transportation MVPA. Higher age and female gender respondents had lower levels of recreational MVPA, while trail users and those who perceived favorable indoor recreational access had higher levels of recreational MVPA.

**Conclusions:**

PA primarily occurred in the occupational domain among this sample of rural mid-Western adults. Findings highlight the need for multilevel interventions to address PA across multiple domains in rural communities, especially among females and older adults.

## Background

Physical activity (PA) has myriad benefits including improved weight status, reduced risk of various cancers and cardiovascular disease [1]. Most Americans do not meet the guidelines of at least 150-minutes of moderate intensity PA per week, 75-minutes of vigorous intensity PA per week, or a combination of the two [2]. Further, fewer rural residents meet PA guidelines when compared to their urban/suburban counterparts [3]. The PA divide between rural and urban areas should be delineated in order to reduce health disparities and improve health for all.

Physical activity is a complex behavior with a multitude of influences, and is strongly influenced by environmental and policy influences. Multilevel factors encourage engagement in PA such as the environment (e.g., parks, trails, sidewalks [4]) and social support (e.g., friends, family [5]) in rural communities. Ecological models of health behaviors suggest targeting multiple levels of influence (e.g., individual, interpersonal) [6]. Intervening at multiple levels takes into consideration both the individual and the environment in which the individual resides.

In 2016, a specific call to action for rural active living promoted the application of a refined ecological approach to determine the correlates of domain-specific PA in rural communities [7]. Further, the call urges the development of evidence-based interventions to improve PA in rural communities. Recent community-based, multilevel trials have been implemented in rural areas – with limited success [8, 9]. In particular, applications of multilevel trials geared toward PA improvement in rural communities often encounter issues due to small sample sizes and implementation challenges inherent to rurality (e.g., culture) [8]. Additionally, many multilevel trials tend to focus on increasing weekly total PA as the outcome of interest, with special attention paid to leisure-time PA – especially in high-income countries [10]. However, other domains of PA in addition to leisure-time PA provide opportunities for individuals from rural communities to meet the recommended guidelines. The four common domains of PA include occupational, household, transportation-related, and recreational [11–14]. Several studies have examined multilevel correlates of domain-specific PA in urban/suburban communities [15]; however, less is understood about how these domains manifest in rural areas. One study of rural Midwestern adults found the domains of active living (i.e., walking and biking) and sport (i.e., athletic activities and exercises) had domain-specific correlates in policy, neighborhood characteristics, and social support among rural Midwestern adults while the domains of occupational and house work PA did not [5]. In the domains of active living and sport, rural residents were more likely to engage in active living if they agreed with government funding for PA resources, and they were more likely to engage in sport if they had social support to exercise [5].

In another study among rural Midwestern adults, unique correlates were found for the previously discussed domains of PA and other domains such as active transportation, yard work in conjunction with housework, and recreation [16]. The results further demonstrated the social and environmental correlates of PA are specific to the domain being measured. For example, female participants have been socialized to engage in more PA through housework when compared to male participants, while male participants have been socialized to engage in more leisure-time PA when compared to single female participants [16]. Additionally, the authors assessed correlates of intensity-specific PA and found greater awareness and use of PA-related resources encouraged engagement in vigorous PA [16].

The literature is rich in evidence supporting the impact of individual and environmental level correlates of recreational and overall PA, yet lacks in providing evidence in the context of rural communities. Furthermore, the understanding of individual and environmental level correlates of domain-specific PA in rural communities remains limited. Therefore, to better understand the correlates of overall and domain-specific (i.e., transportation, occupational, and recreational) MVPA in rural communities in order to inform PA interventions, the objectives of the present study were to (1) provide estimates of overall and domain-specific total weekly MVPA, and (2) examine associations of individual and environmental factors with overall and domain-specific weekly MVPA minutes.

## Methods/design

### Participants and procedures

The current study used baseline data collected from August 2019 to September 2020 from a sample of rural community members participating in a physical activity intervention across a rural Midwestern region. Adult participants who were able to be physically active were recruited by address-based sampling, referral, and community outreach across 14 rural communities in the region – more detailed information on sampling and study eligibility can be found elsewhere [9]. Rurality was determined by using the Rural-Urban Continuum Codes (RUCC) where one equates to the most urban and nine equates to the most rural [17]. Prior to data collection, research assistants obtained informed consent from participants. Baseline measures were collected for a full baseline sample of 1252 participants by trained research assistants via telephone survey. This study was approved by the Institutional Review Board of the sponsoring institution (#201809089).

### Measures

#### Physical activity outcomes

Physical activity (PA) was assessed using the Global Physical Activity Questionnaire (GPAQ) [18, 19]. The instrument assesses PA overall and across three domains including occupational, transportation, and recreation. The GPAQ has demonstrated moderate validity as compared to accelerometry with respect to moderate to vigorous PA [18].

#### Individual

Participants provided demographic information including age, gender, education, race/ethnicity, and income. Age was operationalized according to the PA Guidelines for Americans (Adults aged 18–64, Older adults aged 65+). Gender and education were dichotomized into male or female and high school degree or less and greater than a high school degree. Race was also dichotomized into white or non-white and income of less than $50,000 or greater than $50,000 via median split, respectively. Individual characteristics related to physical activity behavioral factors were also collected and included trail use. Trail use was characterized by participant’s reporting having used their local trail or not.

#### Environmental

Three perceived neighborhood environment subscales were used from the abbreviated Neighborhood Environment Walkability Scale (NEWS), along with the Rural Active Living Perceived Environment Support Scale (RALPESS) [20, 21]. The NEWS subscale used characterizes safety from traffic (five items, Cronbach’s α = 0.74) [20]. RALPESS subscales used included indoor recreational access (six items, Cronbach’s α = 0.91), as well as the area around the home (five items, Cronbach’s α = 0.79) [21]. Negative statements were reverse coded, and items were averaged to compute scores for each subscale. Response options for each item ranged on a four-point Likert scale (1 = “strongly disagree” to 4 = “strongly agree”).

### Analysis

Descriptive statistics including means and frequencies were captured for all variables of interest (i.e., physical activity outcomes, individual and environmental factors). To account for missing data from variables of interest, multiple imputation was performed using multivariate imputation by chained equations [22]. Additionally, due to the non-normal distribution of the data, generalized linear models with negative binomial error distributions were used examining the relation between self-reported age, gender, education, race, trail use, and perceptions of the neighborhood environment (i.e., safety from traffic, indoor recreational access, and around the home environment), with domain-specific (i.e., occupation, transportation, recreation) and overall weekly MVPA minutes. All analyses were conducted using STATA (version 15).

## Results

**Fig. 1:**
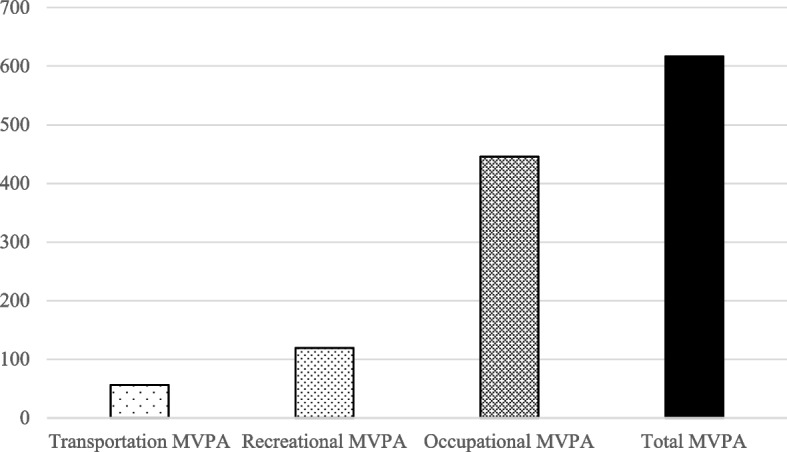
Mean weekly minutes of MVPA by domain. *Overall MVPA*

The analytical sample dropped from 1252 participants to 1241 participants due to relevant missing data. The sample (mean age = 57.0 years, SD = 16.7 years) was predominantly female and white (Table 1). When examining education levels of the sample, 33.5% had a high school diploma or lower. Overall, participants reported engaging in an average of 616.9 (SD = 1141.5) minutes of weekly MVPA. When broken down further by domain, occupational MVPA contributed the most weekly minutes (mean = 445.8, SD = 1024.6), followed by recreational MVPA (mean = 119.5, SD = 222.6), and lastly transportation related MVPA (mean = 56.4, SD = 209.9), see Fig. 1.

**Table 1:** Characteristics of Rural Adults (*N* = 1241), *Heartland Moves*, Southeast Missouri, 2019–2020

Characteristic	Mean (SD) or %
**Individual**	
*Demographics*	
Age, mean (SD [range])	57.0 (16.7 [19–96])
Gender (Female), %	67.8%
Education (≤ High School Diploma), %	33.5%
Race (non-White), %	16.2%
*Physical Activity Behavioral Factors*	
Trail Use, %	56.6%
**Perceived Neighborhood Environment**	
Indoor Recreational Access, mean (SD)	3.2 (0.6)
Area Around Home, mean (SD)	2.4 (0.6)
Safety From Traffic, mean (SD)	2.7 (0.6)
**Physical Activity**	
Meets PA Guidelines	58.5
Overall Weekly Minutes of MVPA, mean (SD)	616.9 (1141.5)
Occupational Weekly Minutes of MVPA, mean (SD)	445.8 (1024.6)
Transportation-related Weekly Minutes of MVPA, mean (SD)	56.4 (209.9)
Recreational Weekly Minutes of MVPA, mean (SD)	119.5 (222.6)

When examining individual and environmental correlates of overall weekly MVPA, only individual correlates, including age, gender, and education were statistically significant (Table 2). Compared to younger adults, older adults’ overall weekly MVPA minutes lowered by 58% (95% CI = 48, 66%) with all other variables held constant. When compared to males, females had 35% (95% CI = 22, 46%) fewer weekly minutes of overall MVPA. Finally, when compared to those of higher education, participants with lower education levels had 44% (95% CI = 16, 79%) more weekly minutes of overall MVPA.

**Table 2:** Multivariate associations of individual and perceived environmental level factors with overall and domain specific weekly MVPA minutes in rural Midwestern adults, (*N* = 1241)

	Overall MVPA	Occupational MVPA	Transportation MVPA	Recreational MVPA
	Odds Ratio (95% CI)			
**Individual factors**				
*Demographics*				
Age (65+)	**0.41 (0.34–0.52)**	**0.31 (0.23–0.42)**	0.72 (0.46–1.12)	**0.76 (0.58–0.99)**
Gender (Female)	**0.65 (0.54–0.78)**	**0.63 (0.49–0.81)**	**0.63 (0.42–0.94)**	**0.59 (0.47–0.75)**
Education (≤ High School Diploma)	**1.44 (1.16–1.79)**	**1.43 (1.08–1.89)**	**2.09 (1.34–3.26)**	0.90 (0.68–1.19)
Race (non-White)	1.06 (0.82–1.38)	1.00 (0.69–1.45)	1.30 (0.85–2.00)	1.43 (0.99–2.08)
*Physical Activity Behavioral Factors*				
Trail Use	1.23 (0.99–1.53)	1.03 (0.77–1.39)	**1.60 (1.02–2.49)**	**1.88 (1.44–2.44)**
**Perceived environmental factors**				
Indoor recreational access	1.01 (0.83–1.22)	0.92 (0.72–1.18)	**0.59 (0.38–0.92)**	**1.42 (1.07–1.89)**
Safety from traffic	0.99 (0.83–1.19)	0.96 (0.76–1.22)	0.73 (0.48–1.12)	1.11 (0.91–1.36)
Area around home	0.99 (0.83–1.19)	0.98 (0.80–1.20)	1.33 (0.97–1.83)	1.04 (0.86–1.27)

Models control for clustering effects of town. Bold indicates a statistically significant association at *p* < .05

### Occupational MVPA

When examining individual and environmental correlates of occupational PA, the significant associations remained the same as for overall MVPA, with no environmental correlates being statistically significant (Table 2). For older adults, participant’s occupational weekly MVPA minutes lowered by 69% (95% CI = 58, 77%) with all other variables held constant, when compared to younger adults. When compared to their male counterparts, female participants had 37% (95% CI = 19, 51%) fewer weekly minutes of occupational MVPA. Finally, when compared to those of higher education, participants with lower education levels had 43% (95% CI = 8, 89%) more weekly minutes of occupational MVPA.

### Transportation-related MVPA

When examining individual and environmental correlates of transportation-related MVPA, both individual (i.e., gender, education, and trail use) and environmental (i.e., perceived indoor recreational access) correlates were statistically significant (Table 2). When compared to their male counterparts, female participants had 37% (95% CI = 6, 58%) fewer weekly minutes of transportation MVPA. When compared to those of higher education, participants with lower education levels had 109% (95% CI = 34, 226%) more weekly minutes of transportation MVPA. When compared to non-trail users, participants who used trails had 60% (95% CI = 2, 149%) more weekly minutes of transportation MVPA. Finally, when considering environmental correlates of recreational MVPA, for every unit increase in perceived indoor recreational access, participant weekly minutes of transportation MVPA decreased by 41% (95% CI = 8, 62%).

### Recreational MVPA

When examining individual and environmental correlates of weekly recreational MVPA minutes, both individual (i.e., age, gender, and trail use) and environmental (i.e., perceived indoor recreational access) correlates were statistically significant (Table 2). For older adults, participant’s recreational weekly MVPA minutes lowered by 24% (95% CI = 1, 42%) with all other variables held constant, when compared to younger adults. Compared to males, females had 41% (95% CI = 25, 53%) fewer weekly minutes of recreational MVPA. When compared to non-trail users, participants who used trails had 88% (95% CI = 44, 144%) more weekly minutes of recreational MVPA. Finally, when considering environmental correlates of recreational MVPA, for every unit increase in perceived indoor recreational access, participant weekly minutes of recreational MVPA increased by 42% (95% CI = 7, 89%).

## Discussion

This study was among the first to elucidate multilevel correlates of overall and domain-specific PA in a rural population. The findings of the study demonstrate the influence of age, gender, race, and education on overall and domain-specific PA. Further, this sample of rural residents had high levels of occupational PA and conversely, low levels of transportation PA. Findings from this study contribute to the gap in literature regarding multilevel correlates of domain-specific PA in rural communities, and will benefit the design and adaptation of interventions seeking to improve levels of domain-specific PA, ultimately reducing adverse health outcomes, among rural community members.

Consistently across the domains and overall PA, increased age was found to be associated with decreasing PA. PA is well known to decrease as age increases across multiple domains [10]. The transportation domain was the one domain in which age did not reduce PA. Transportation PA in rural areas is limited due to the disperse nature of rural communities, low population density, and ample parking [23–26]. These rural communities are car-centric as the walkability and connectivity are low, and various destinations of interest (e.g., pharmacy, grocery store) are often not near one’s home, or are located in a different community. Therefore, it is likely since all ages depend on automobiles for transportation, transportation PA did not decrease with age. While some have found maintenance of leisure and recreational activities among older adults [27], we found an inverse relationship between recreational PA and age.

In addition to being influenced by age, overall MVPA and every MVPA domain were associated with various individual level correlates. In particular, the gender of a participant determined the extent to which a participant engaged in overall MVPA and each MVPA domain. The trend maintained consistency across the different MVPA outcomes, where female participants engaged in significantly fewer weekly minutes of overall MVPA, occupational MVPA, transportation MVPA, and recreational MVPA than male participants. In general, women are known to be less physically active than their male counterparts in rural areas [1, 12, 16, 28]. Although the gender divide in MVPA has been empirically noted previously, research has been limited to overall MVPA and the recreational domain of MVPA.

Our findings highlight a significant difference in weekly minutes of occupational and transportation MVPA in addition to overall and recreational MVPA based on gender. Females in this sample of rural communities engaged in fewer minutes of weekly recreational MVPA than males, a finding aligning with previous conclusions of gender differences in the recreational MVPA domain in rural communities [16]. Unlike the age variable, a difference by gender was detected in the transportation MVPA domain. Due to the disperse layout of rural communities, walking or cycling to destinations in rural communities is uncommon [7, 29]; however, providing an environment conducive to transportation MVPA, for example, sidewalks in good repair leading to points of interest, may aid in increasing MVPA in rural communities, especially among female residents. Our findings reveal the importance of designing PA domain-specific interventions to account for gender disparities in MVPA, particularly in rural areas.

Educational attainment and PA have historically shown a direct relationship [11, 12, 30–32]; however, the current study demonstrated an inverse relationship between education and PA, specifically in the overall, occupational, and transportation domains. The occupational domain was the main driver of overall MVPA in this study, likely due to more manual labor job participation (e.g., manufacturing, construction) leading to more occupational MVPA, and subsequently, overall MV|PA. Higher education is oftentimes not a prerequisite for manual labor jobs, therefore, it is also feasible those with lower education could not afford a vehicle and associated costs (e.g., fuel, insurance, maintenance). Therefore, they may have relied on other modalities (i.e., walking, cycling) to get to their destination – thereby increasing their transportation MVPA [31].

Lastly, trail use and perceived indoor recreation access were predictive of weekly minutes of recreational and transportation MVPA among rural community members. More walking trail use has been associated with more weekly total PA [4]; however, this study contributes to the literature by demonstrating the domain-specific impact of trail use on weekly minutes of MVPA among participants from rural communities in the Midwest. Perceived indoor recreation accesses association with improved PA has been noted previously [33, 34]; however, the current finding is specific to rural residents and the recreational domain of MVPA. Further, as indoor recreational access perceptions increased, transportation MVPA went down. The finding of increased recreational access and decreased transportation MVPA may mean the car-centric rural area residents were inclined to drive to local facilities (e.g., gyms, recreation centers) to exercise. By raising awareness about the availability and proximity to local trails and recreation facilities, individuals and families in rural communities may attain more minutes of recreational PA and improve their personal health outcomes as well as the health of their communities.

### Limitations

The cross-sectional nature as well as the self-reported variables limit the ability to distinguish causality. This study took part in a specific geographic region of the U.S. thereby limiting the generalizability of the findings. Not all levels of the socioeconomic model (e.g., social support) were measured and analyzed in this study. Future analyses should include the measurement and subsequent analysis of other important covariates. There was a lack of diversity in the study sample; however, the sample demographics were aligned with regional demographics. Lastly, the PA data was self-reported, which can be overestimated.

Despite the limitations, this study has several strengths. The study attained a relatively large sample size containing respondents of the baseline survey, especially considering the sample consisted of rural community members. The fact that the study focused analyses on participants from rural communities in particular represents another strength, due to the frequent under-representation of the rural demographic in PA research and in human subjects research in general. Lastly, the study revealed preliminary evidence for the individual and environmental correlates of not just overall PA, but also domain-specific PA in rural communities to identify potential targets for future interventions seeking to improve PA in rural communities.

## Conclusion

It is imperative, when measuring PA, to include the domain of the activity along with a broad array of contextual variables. Commonly, PA is viewed as an overall concept where various domains of PA are summed to create a dichotomous variable of meeting guidelines; however, actionable information may be lost when coalescing PA across domains. Physical activity comes in many forms and this study found occupational and recreational PA as important domains for rural people. By examining multilevel correlates of specific domains of PA, we can have a deeper understanding on how to best promote physical activity for different groups in different environments. Ultimately, more targeted interventions could help increase PA and reduce chronic disease at the population level in rural communities.

## Data Availability

The data supporting the conclusions of this article are available from the corresponding author on reasonable request.

## Abbreviations

CI: Confidence Interval
GPAQ: Global Physical Activity Questionnaire
NEWS: Neighborhood Environment Walkability Scale
MVPA: Moderate to Vigorous Physical Activity
PA: Physical Activity
RALPESS: Rural Active Living Perceived Environment Support Scale
RUCC: Rural-Urban Continuum Code
SD: Standard Deviation
